# Assessment of the Orbit® subretinal delivery system (Orbit® SDS device) and prototypes in adult and juvenile canine eyes

**DOI:** 10.1007/s13346-025-01929-2

**Published:** 2025-08-02

**Authors:** Jennifer C. Kwok, Alexa P. Gray, Yu Sato, Svetlana Savina, Natalia Dolgova, Raghavi Sudharsan, Sanford L. Boye, Shannon E. Boye, Sam Reichenbacker, Tom Meyer, Kirsten Stoner Cummiskey, William A. Beltran

**Affiliations:** 1 Division of Experimental Retinal Therapies, Department of Clinical Sciences & Advanced Medicine, School of Veterinary Medicine, University of Pennsylvania, Philadelphia, USA; 2 Department of Pediatrics, University of Florida, Gainesville, FL USA; 3 Genentech, Inc, San Francisco, USA; 4 Novartis Pharmaceuticals Corporation, East Hanover, USA

**Keywords:** Orbit^®^ SDS device, Subretinal injection, Delivery, Suprachoroidal space, AAV, Canine

## Abstract

This study aimed to assess the applicability of the Orbit^®^ Subretinal Delivery System [Orbit^®^ SDS, 2nd Generation, clinical-grade device] and prototypes for subretinal injection (SRI) in dogs of different ages. Following ex vivo and in vivo testing of the clinical grade Orbit^®^ SDS in 7 adult canine eyes, 8 normal dogs (13 eyes; age range: 10–72 weeks) were injected with an AAV2/5-CBA-tdTomato-WPRE vector using different Orbit^®^ SDS prototype devices. Dogs underwent non-invasive fundus photography regularly post-injection (PI). Retinal imaging including optical coherence tomography (OCT) angiography (OCTA) and *en face* null OCT was performed in most dogs at 5- and 9-weeks PI. Following termination, eyes were examined histologically. Ex vivo and in vivo SRI could be performed in 7 adult eyes including in that of a dog with end stage retinal degeneration. SRI of AAV2/5-CBA-tdTomato-WPRE was also successful in all 13 eyes including those of juvenile animals, yet this frequently required multiple attempts and/or using custom-modified devices that were adjusted to the smaller curvature of the ocular globes. Localized tdTomato expression was found in 11/13 eyes along the cannula path in the suprachoroidal space (SCS) in the sclera and choroid. Clinical and cellular signs of inflammation were seen in 7/13 eyes. The Orbit^®^ SDS device allows for controlled subretinal injections via a posterior approach in adult and juvenile dogs. There was variable vector reflux from the subretinal bleb and/or leakage from the device into the SCS. A cellular immune reaction to the AAV capsid/transgene may have contributed to retinal and choroidal inflammation.

**Supplementary Information:**

The online version contains supplementary material available at 10.1007/s13346-025-01929-2.

## Introduction

Access to the subretinal space (SRS), which is located between the neuroretina and the retinal pigment epithelium (RPE) is used by vitreoretinal surgeons to inject tissue plasminogen activator (TPA) for the treatment of submacular hemorrhages of different etiologies, or balanced salt solution (BSS) for the repair of full-thickness macular hole [1]. The recent advent of gene and cell therapies for age-related macular degeneration (AMD) and inherited retinal diseases have also made subretinal injections (SRI) the primary method by which photoreceptors and the retinal pigmented epithelium (RPE) can be targeted for the treatment of these blinding conditions in clinical practice and preclinical studies [1–5].

The most common method to deliver suspensions or solutions (e.g. viral vectors, cells, tissue plasminogen activator) into the subretinal space is via an anterior approach (a.k.a. transvitreal approach) whereby a needle is first inserted into the vitreal cavity through a transscleral trocar placed at the level of the pars plana. In human patients, pars plana vitrectomy is generally performed prior to transvitreal SRI, with some surgeons opting for internal limiting membrane (ILM) peeling as well to facilitate retinal perforation [6]. In preclinical studies in rodents, puncture of the cornea and advancement of the needle into the vitreal cavity after displacement of the lens is most commonly used [7] while in larger animal models the entry into the vitreal cavity occurs at the level of the pars plana [8]. Advancement of the needle at the end of which there is a thin (38–41 G) cannula results in a focal puncture of the retina (= retinotomy) through which a solution/suspension can be injected into the SRS creating a localized retinal detachment (= retinal bleb). Surgical methods to access the SRS via a posterior transscleral approach have also been developed, however they provide limited visualization of the fundus at the time of the injection [9, 10].

Transvitreal SRI is not without potential challenges. Intra-operative risks include partial or significant reflux of the solution/suspension into the vitreal cavity that can possibly contribute to the triggering of an immune reaction as the vitreal cavity is less of an immune-privileged site than the SRS. There is also a potential for a large retinotomy to be created from lateral hand movements during injection [11]. In addition, if a subretinal bleb is created too quickly with too high of an injection pressure, retinal cells can also undergo stress [12] which can potentially result in decreased cell function [13]. This is of particular concern when higher injection pressures may be required to initiate and propagate subretinal blebs depending on patient age or condition [14]. Post-operative complications associated specifically with transvitreal SRI include retinal tears, panuveitis with persistent retinal detachment [15], and formation of choroidal neovascularization (CNV) lesions [16]. These challenges in transvitreal SRI indicate a value for an alternative approach to subretinal delivery of therapies.

The posterior transscleral or suprachoroidal approach for SRI was first described by Parikh et al. [9] in the rodent model. A sclerotomy is performed before introducing a Hamilton^®^ needle that is inserted through the choroid to access the subretinal space in a free-handed manual injection approach.

More recently, the Orbit^®^ SDS device was developed to provide precise, targeted subretinal delivery via a suprachoroidal approach thus circumventing the need for a prior vitrectomy, and a retinotomy. By preventing reflux of subretinally-injected material into the vitreal cavity, it was designed with the goal of reducing surgical complications/inflammation associated with transvitreal SRI [17]. A flexible cannula introduced through a 3-mm wide sclerotomy into the suprachoroidal space (SCS) conforms to the curvature of the eye. Once the cannula is advanced to the desired injection location, a needle advancement knob allows for controlled extensions of a 35G curved microneedle. Separate fluid lines allow for delivery of both BSS and the drug solution/suspension with minimal mixing (Suppl. Figure 1). The Orbit^®^ SDS device was initially developed for cell therapy delivery [18, 19], and has been recently used in a Phase I/II clinical trial (NCT03846193) aimed at evaluating the safety, dose response, and efficacy of an AAV-based gene therapy product (GT005) that encodes for a human complement factor in people with geographic atrophy secondary to AMD.

The dog with its large sized eye [20], its cone-rich *area centralis* that has a peak density of cone photoreceptors in its fovea-like area [21, 22], and its predisposition to inherited retinal degenerations [23] provides a valuable model system to test novel ocular drug delivery devices. To this date, subretinal delivery of gene and cell therapy products to the subretinal space of the canine eye has been achieved via an anterior/transvitreal approach [8, 24, 25]. In this study we evaluated the ability to perform subretinal injections with Orbit^®^ SDS devices in adult and juvenile dogs, assessed the potential reflux of viral vector into the suprachoroidal space, and began to evaluate the safety profile of this route of subretinal injection when used to deliver AAV vectors.

## Materials and methods

### Animals

A total of 14 animals (20 eyes) including 4 beagles and 10 middle-sized mongrel dogs were used for this work. In a pilot study, both freshly enucleated eyes from an adult wildtype (WT) dog were used to test the feasibility of subretinally injecting BSS with the Orbit^®^ SDS clinical-grade device (2nd generation design, FDA 510(k) cleared). Subsequently, the same type of device was used in one eye of 4 adult WT and 1 adult *PDE6B*^−/−^ mutant dog [26] to deliver an AAV2/5 vector carrying the green fluorescent protein (GFP) reporter transgene under control of the photoreceptor specific G-protein coupled receptor kinase 1 (GRK1) promoter (AAV2/5-GRK1-*GFP*). The single adult *PDE6B*^−/−^ mutant dog included in this study was used to begin assessing whether the subretinal space could be accessed with the Orbit^®^ SDS clinical-grade device when attempting an injection in a retina with advanced degeneration [27]. Finally, in another series of 8 normal dogs (13 eyes) with an age ranging from 10 to 72 weeks, subretinal injections of an AAV2/5 vector carrying the tdTomato cDNA and a Woodchuck Hepatitis Virus Posttranscriptional Regulatory Element (WPRE) under control of the ubiquitous chicken β-actin (CBA) promoter were attempted with either the Orbit^®^ SDS clinical-grade device or one of five custom-made Orbit^®^ SDS prototype devices, including one developed for injecting non-human primates (NHPs) (Table 1). Juvenile dogs (as young as 10 weeks of age) were selected in this study for two translational reasons: (1) to determine whether subretinal injections with an Orbit^®^ SDS device could be performed at ages that correspond to the onset of photoreceptor loss in several naturally-occurring canine models of inherited retinal degeneration [28–30], and (2) to test this subretinal approach at an age when the canine eye has a similar size to that of the human neonate [20, 31].

**Table 1:** Summary of surgical outcomes in dogs with Orbit^®^ SDS and prototypes

Dog ID	Genotype	Age at SRI (wks)	Eye	Orbit^®^ SDS (device type)	Surgical Summary	AC paracentesis	SR hemorrhage	Retinotomy suspected intraoperatively
N290	WT	320	OD	Orbit^®^ SDS 2nd Gen	Successful if sclerotomy ∼ 8–10 mm post. to limbus	No	N/A	No
			OS	Orbit^®^ SDS 2nd Gen	Successful in both tapetal and non-tapetal fundus	No	N/A	No
CKCCPG	WT	129	OD	Orbit^®^ SDS 2nd Gen	Successful on 1st needle advancement	No	No	Yes
CKCCRD	WT	128	OD	Orbit^®^ SDS 2nd Gen	Successful on 2nd needle advancement	No	No	Yes
CKCCRY	WT	129	OD	Orbit^®^ SDS 2nd Gen	Successful on 2nd needle advancement	No	Yes	No
CKCCPB	WT	129	OD	Orbit^®^ SDS 2nd Gen	Successful on 1st needle advancement	No	No	No
2353	*PDE6B* ^−/−^	78	OD	Orbit^®^ SDS 2nd Gen	Successful on 3rd needle advancement	Yes	No	No
N356	WT	72	OD	Orbit^®^ SDS 2nd Gen	Successful on 6th needle advancement.	Yes	Yes	No
			OS	Orbit^®^ SDS 2nd Gen	Successful on 1st needle advancement. Injection in the non-tapetal fundus.	No	No	No
SSA-14	*cngb3* ^−/+^	61	OD	Orbit^®^ SDS 2nd Gen	Successful on 7th needle advancement, cannula repositioned once.	Yes	Yes	No
			OS	Orbit^®^ SDS 2nd Gen	Successful on 2nd needle advancement. Injection in the non-tapetal fundus.	No	No	No
N360	WT	19	OD	Orbit^®^ SDS 2nd Gen	Successful on 5th needle advancement, cannula repositioned once.	No	Yes	Yes
			OS	Orbit^®^ SDS 2nd Gen	Successful on 8th needle advancement, cannula repositioned once.	No	Yes	Yes
1054106	WT	19	OD	Orbit^®^ SDS Proto4	Failed on 15 needle advancements.	No	No	No
				Orbit^®^ SDS NHP	No attempt (microneedle malfunction).			
				Orbit^®^ SDS 2nd Gen	Successful on 1st needle advancement. Injected in the non-tapetal fundus.			
N361	WT	19	OD	Orbit^®^ SDS Proto2	Successful on 1st needle advancement.	No	Yes	Yes
			OS	Orbit^®^ SDS Proto3	Failed after 5 needle advancements.	No	Yes	Yes
				Orbit^®^ SDS Proto1	Successful on 2nd needle advancement.			
1168382	WT	12	OD	Orbit^®^ SDS Proto1	Successful on 1st needle advancement.	Yes	Yes	Yes
1180608	WT	10	OD	Orbit^®^ SDS Proto1	Successful on 1st needle advancement.	Yes	Yes	Yes
1180560	WT	10	OD	Orbit^®^ SDS Proto1	Successful on 1st needle advancement.	No	Yes	Yes
			OS	Orbit^®^ SDS Proto2	Successful on 1st needle advancement.	Yes	Yes	Yes

All experiments were performed in accordance with the University of Pennsylvania’s IACUC policies (host institute IACUC# 803254) and recommendations by the Association for Research in Vision and Ophthalmology (ARVO)’s Resolution on the Use of Animals in Ophthalmic and Vision Research. All dogs were assessed to be healthy prior to commencing any experimental procedures.

### Subretinal injection via Orbit® subretinal delivery system (Orbit® injection in ex vivo canine SDS device)

All dogs older than 12 weeks of age were premedicated using 0.25 mg/kg subcutaneous injection of acepromazine (Boehringer Ingelheim, Germany) approximately 30 min prior to induction with intravenous (IV) injection of propofol to effect (Zoetis, USA). Dogs younger than 12 weeks of age were given 0.01–0.02 mg/kg IV or intramuscular (IM) buprenorphine (Par Pharmaceutical, USA) just prior to induction. Anesthesia was maintained on inhalational isoflurane (Isospire™, Dechra, USA) with continuous infusion of IV fluids.

A total of 6 different Orbit^®^ SDS devices were tested in a series of experiments (Table 1). The device was connected to two syringes for injecting solutions - one for BSS and one for viral vector (Suppl. Figure 1). The viral vector solution was drawn into a 0.3 mL Zero Residual™ syringe (SJJ Solutions, Netherlands) to minimize dead volume loss.

**Fig. 1:**
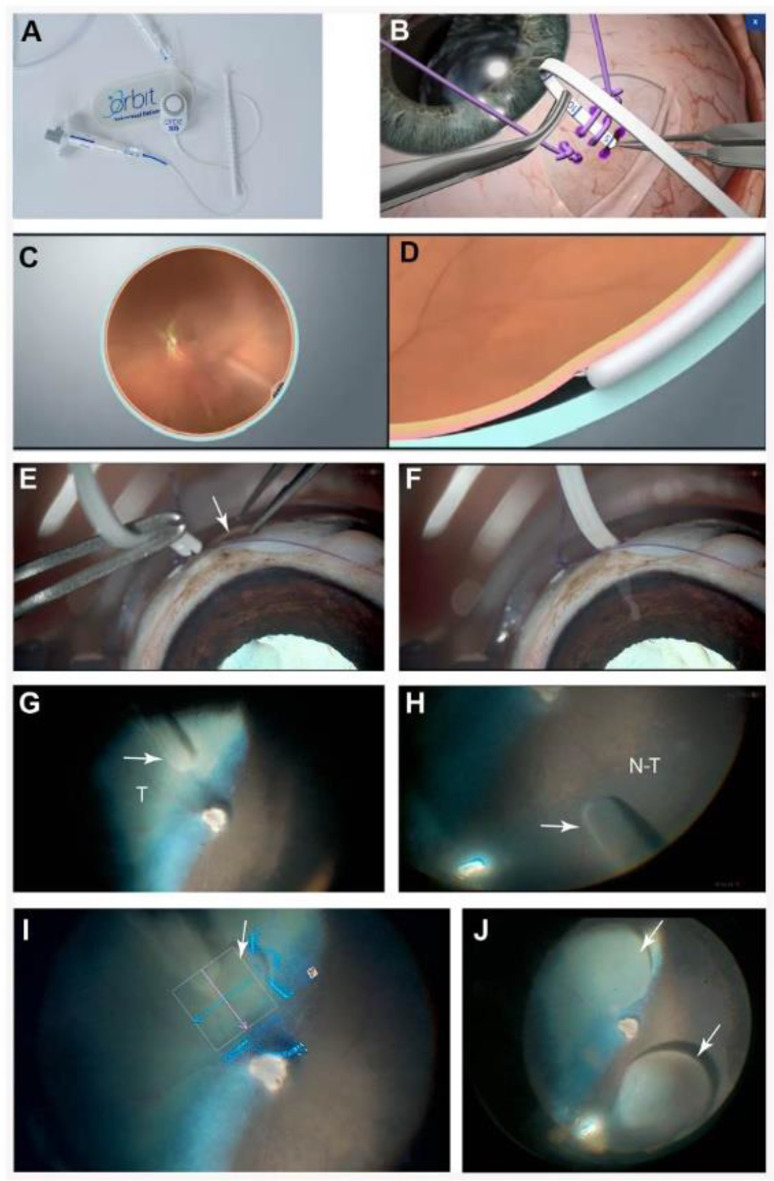
Successful subretinal injection in ex vivo canine eye(s) with the Orbit^®^ SDS device. (**A**) Components of the device. (**B**) Schematic in a human eye showing placement of the cannula into the suprachoroidal space through the sclerotomy site. (**C**) Schematic showing the fundus view of a human eye with the Orbit^®^ SDS cannula introduced into the suprachoroidal space. (**D**) Extension of the curved needle used to penetrate the subretinal space. (**E**-**J**) Ex vivo surgical procedure in an adult WT canine eye. (**E**-**F**) Introduction of the device’s cannula through a 3 mm wide sclerotomy performed ~ 10 mm posterior to the limbus and advancement into the suprachoroidal space. (**G**-**H**) Direct visualization of the progression of the cannula with the surgical microscope allows proper placement of the cannula (white arrows) to the intended targeted delivery site in both tapetal (T) and non-tapetal (N-T) areas. (**I**) Visualization of the needle and formation of an initial 10 µL subretinal bleb (white arrow) with BSS using the intraoperative OCT modality of the surgical microscope. (**J**) 100 µL subretinal blebs (white arrows) formed in the central retina of both tapetal and non-tapetal regions of the canine fundus

A magnetic pad adhered to surgical drapes was used to secure the position and orientation of the Orbit^®^ SDS device. This was placed on surgical drapes over the dog’s forehead, close to the palpebral fissure. Once the sclera was exposed, a scleral stamp provided in the Orbit^®^ SDS kit was used to mark the sclera with surgical ink (Securline^®^, Aspen Surgical Products, Inc., MI, USA). Depending on the age of the dog or eye size, it was positioned 0–4 mm posterior to the limbus to mark the locations of the scleral stay sutures (6 − 0 Vicryl™, Ethicon US, LLC., USA) and the site of the sclerotomy that was ~ 6–10 mm posterior to the limbus to avoid the large scleral venous plexus of the canine eye. The sclerotomy site was cauterized prior to sclerotomy with an 18G Wet-Field^®^ Eraser^®^ cautery (Beaver-Visitec Int. Inc., USA). After making a 3 mm-wide scleral incision with a 1.25 mm angled mini-crescent knife (Sharpoint™ Sharptome™, Surgical Specialties Corp.™, Mexico), any remaining scleral fibers were broken using a straight Sinskey hook until the choroid was visible. The Orbit^®^ SDS cannula was then inserted 5–7 mm through the sclerotomy into the SCS. While visualizing the fundus with a wide-angle lens and chandelier lighting, the cannula was moved to the desired location for injection. The microneedle was advanced by rotating the knob on the main body of the device until it was seen entering the SRS. Using the foot pedal to control the vitrectomy machine that was connected via its VFI line to the syringe containing BSS, a small bleb was first made to confirm needle entry into the SRS. Then, the viral vector was injected manually over a period of approximately 3 min followed by a ten second hold prior to removal of the Orbit^®^ SDS cannula from the eye (Fig. 1). In the case of inadvertent retinal puncture during needle extension, the needle was retracted into the SRS prior to injecting BSS. Ease of access was graded for each device based on the number of needle advancements required to create a subretinal pre-bleb as follows, 1–2 needle extensions, Grade 1; 3–5 needle extensions, Grade 2; >5 needle extensions Grade 3; no bleb formed, Grade 4. Delivery success was defined as successful delivery of viral vectors to the subretinal space. Aqueous humor paracentesis was performed in 6 of the injected eyes due to perceived elevations in intraocular pressure immediately after surgical completion as infusion was not used during any procedure.

All dogs were placed on a 5-week medication course. On the day of injection, dogs were given topical 0.03% flurbiprofen (Amici Pharmaceuticals, USA), 0.3% gentamicin sulfate (Alcon Laboratories Inc., USA), 1% atropine sulfate (Apotex Inc., Canada), 1% tropicamide (Somerset Therapeutics LLC, USA) and 10% or 2.5% phenylephrine (Lifestar Pharma LLC, India). Immediately after injection, neomycin-polymyxin-dexamethasone ointment (Bausch & Lomb Inc., USA) was applied once in injected eyes. Topical 1% atropine ointment (Med-4Vet, USA) was continued once daily in injected eyes for the first week following SRI. Topical 1% prednisolone drops (Alcon Laboratories Inc., USA) were given to injected eyes twice daily for the first two weeks post-injection, then once daily for the following two weeks. Oral prednisone (Amneal Pharmaceuticals LLC, USA) was given at a dose of 1 mg/kg twice daily for the first three weeks, then once daily during the fourth week. All dogs were given amoxicillin (Clavacilin™, Dechra, USA) orally twice daily for 5 weeks. A subconjunctival injection of 4 mg of triamcinolone acetonide (Amneal Pharmaceuticals LLC, USA) was given on the day of injection and at the beginning of the 5th week of postoperative medications. In 6 dogs, oral prednisone was given at 1 mg/kg twice daily for 3 days prior to SRI as a measure to prevent inflammation.

### Confocal scanning laser ophthalmoscopy and optical coherence tomography

Imaging was performed at 5 weeks and 9 weeks post-injection (PI) using a Spectralis^®^ HRA-OCT2 unit (Heidelberg Engineering Inc, Germany). Confocal scanning laser ophthalmoscopy (cSLO) images were taken in infra-red (IR), blue light autofluorescence and red-free imaging, followed by spectral domain optical coherence tomography (SD-OCT) to obtain sequential images. At each imaging session, dogs were placed under general anesthesia using the above-mentioned protocol with the only difference being that buprenorphine was never given. OCT angiography (OCTA) was used to examine SRI sites at each timepoint. At the same time, *en face* OCT was performed at SRI sites or sites of attempted SRI.

### Ophthalmic examinations and fundus photography

All dogs received eye examinations prior to SRI, at 24 h PI until subretinal blebs had reattached, then weekly until termination at 9 weeks PI. Eye examinations included external assessment of the adnexa and general ocular comfort, anterior segment examination by slit lamp and a fundus examination via indirect ophthalmoscopy. Finally, tonometry (iCare Tonovet, Finland) was performed to record intraocular pressures at each eye examination. Eye examinations were always performed after mydriasis is induced with topical 1% tropicamide and/or 2.5–10% phenylephrine. All dogs received fundus photography (RetCam Shuttle^®^, Natus Medical Inc., USA) pre-SRI, immediately after SRI and weekly until termination. Topical 0.5% proparacaine (Bausch & Lomb, USA) was applied as a local anesthetic prior to RetCam fundus photography. The 8 WT dogs injected with AAV2/5-CBA-*tdTomato*-WPRE were imaged for tdTomato expression (Topcon Healthcare, USA) at the 5- and 9-weeks PI timepoints.

### Viral vectors

A viral vector carrying the GFP reporter gene under control of the GRK1 photoreceptor-specific promoter (AAV2/5-GRK1-*GFP*) was injected (0.15 mL, 1.51 × 10^11^ vg/mL) in the right eye (OD) of 4 adult normal dogs (ages ~ 129 weeks), and 1 adult mutant dog (*PDE6B*^*−/−*^ aged 78 weeks) to assess vector targeting of photoreceptors. Subsequently, a tdTomato-expressing AAV vector containing a ubiquitous CBA promoter (AAV2/5-CBA-*tdTomato*-WPRE) was injected in 8 normal dogs of various ages, including 2 adults (ages 61–72 weeks, 4 eyes total) and 6 juveniles (ages 10–19 weeks, 10 eyes total). This vector was injected at the following titers: 1 × 10^12^ vg/mL in 2 eyes, 5 × 10^11^ vg/mL in 2 eyes, 1.5 × 10^11^ vg/mL in 9 eyes and 5 × 10^10^ vg/mL in 1 eye. All dogs were injected with a volume of 0.15–0.18 mL of viral vector, except for a juvenile dog that was injected with 0.1 mL of viral vector (Table 2).

**Table 2:** Summary of all dogs and vectors used

Dog ID	Genotype	Age at SRI (wks)	Eye	Vector	Titer (vg/mL) / Vol (mL)
N290	WT	320	OD	None (BSS only)	0 / 0.10 × 2
			OS	None (BSS only	0 / 0.10 × 2
CKCCPG	WT	129	OD	AAV2/5-GRK1-*GFP*	1.51 × 10^11^ / 0.15
CKCCRD	WT	128	OD	AAV2/5-GRK1-*GFP*	1.51 × 10^11^ / 0.15
CKCCRY	WT	129	OD	AAV2/5-GRK1-*GFP*	1.51 × 10^11^ / 0.15
CKCCPB	WT	129	OD	AAV2/5-GRK1-*GFP*	1.51 × 10^11^ / 0.15
2353	*PDE6B* ^−/−^	78	OD	AAV2/5-GRK1-*GFP*	1.51 × 10^11^ / 0.15
N356	WT	72	OD	AAV2/5-CBA-TdTomato-WPRE	1 × 10^12^ / 0.18
			OS	AAV2/5-CBA-TdTomato-WPRE	1 × 10^12^ / 0.18
SSA-14	*cngb3* ^−/+^	61	OD	AAV2/5-CBA-TdTomato-WPRE	5 × 10^11^ / 0.18
			OS	AAV2/5-CBA-TdTomato-WPRE	5 × 10^11^ / 0.18
N360	WT	19	OD	AAV2/5-CBA-TdTomato-WPRE	1.5 × 10^11^ /0.15
			OS	AAV2/5-CBA-TdTomato-WPRE	1.5 × 10^11^ /0.15
1054106	WT	19	OD	AAV2/5-CBA-TdTomato-WPRE	1.5 × 10^11^ /0.15
N361	WT	19	OD	AAV2/5-CBA-TdTomato-WPRE	1.5 × 10^11^ /0.15
			OS	AAV2/5-CBA-TdTomato-WPRE	5 × 10^10^ /0.15
1168382	WT	12	OD	AAV2/5-CBA-TdTomato-WPRE	1.5 × 10^11^ /0.15
1180608	WT	10	OD	AAV2/5-CBA-TdTomato-WPRE	1.5 × 10^11^ /0.15
1180560	WT	10	OD	AAV2/5-CBA-TdTomato-WPRE	1.5 × 10^11^ /0.10
			OS	AAV2/5-CBA-TdTomato-WPRE	1.5 × 10^11^ /0.10

### Histological evaluation

Dogs were terminated with an injection of pentobarbital sodium euthanasia solution (Pentobarsol™; Dechra, USA) given intravenously at 200 mg/kg, and death was confirmed via cardiopulmonary auscultation by a veterinarian. All globes were fixed in 4 − 2% paraformaldehyde (PFA). A slit was made at the level of the *pars plana* of each globe before it was fixed for 3 h in 4% PFA during which the anterior and posterior segments were separated after the first 45 min. Then, the posterior cups were transferred into 2% PFA for 24 h after which they were trimmed and then placed into 15% and 30% sucrose in PBS for 24 h before embedding in Optimal Cutting Temperature medium. Globes injected with AAV2/5-GRK1-*GFP* were cut along the 4 meridians and sections that encompassed the bleb and bleb transition areas were evaluated for native GFP fluorescence. For globes injected with AAV2/5-CBA-*tdTomato*-WPRE, sections were made perpendicular to the direction of the Orbit^®^ SDS cannula at the time of injection to assess for the presence of native tdTomato fluorescence within the choroid and/or sclera that would indicate potential vector reflux into the SCS. Using the black tapetal scar that marks the site of SRI as a reference, sections were made proximal and distal to the cannula tract (Suppl. Figure 2). These sections were then evaluated for tdTomato labelling and stained with hematoxylin and eosin (H&E) to examine retinal, choroidal, and scleral morphology. To assess cellular inflammation in ocular tissues, indirect fluorescence immunohistochemistry (IHC) was performed using Iba1, CD4, CD8, CD18 and CD20 primary antibodies (Suppl. Table 1), and Hoechst 33,342 nuclear stain (Molecular Probes) was used to label cell nuclei as previously reported [24]. Sections immunolabeled with appropriate fluorescent secondary antibodies (AlexaFluor™, ThermoFisher Scientific) were examined under a widefield epifluorescence microscope (Axioplan, Carl Zeiss Meditec, Jena, Germany). Images were digitally captured with a Zeiss Axiocam camera and imported into a graphics program (Adobe Illustrator, Adobe, Mountain View, CA).

## Results

### Pilot feasibility study – ex vivo injections in adult canine eyes with the Orbit® SDS device

Two freshly enucleated adult canine eyes were used to assess the ability to perform SRI with the clinical-grade Orbit^®^ SDS device (2nd generation design, FDA 510(k) cleared. Resistance to the introduction of the cannula into the SCS was felt when the sclerotomy was performed 6 mm posterior to the limbus (as done in human eyes). In addition, because of the vast intrascleral vascular network (intrascleral plexus) found in canine eyes at that location, the decision was made to perform the sclerotomy more posteriorly (~ 8–10 mm from the limbus). This enabled an easy introduction in the SCS of the cannula that could be pushed in to reach the posterior pole of the eye. Successful injections of 0.1 mL of BSS were achieved in both the tapetal and non-tapetal areas (Fig. 1).

### Targeting photoreceptors in adult WT eyes

**Fig. 2:**
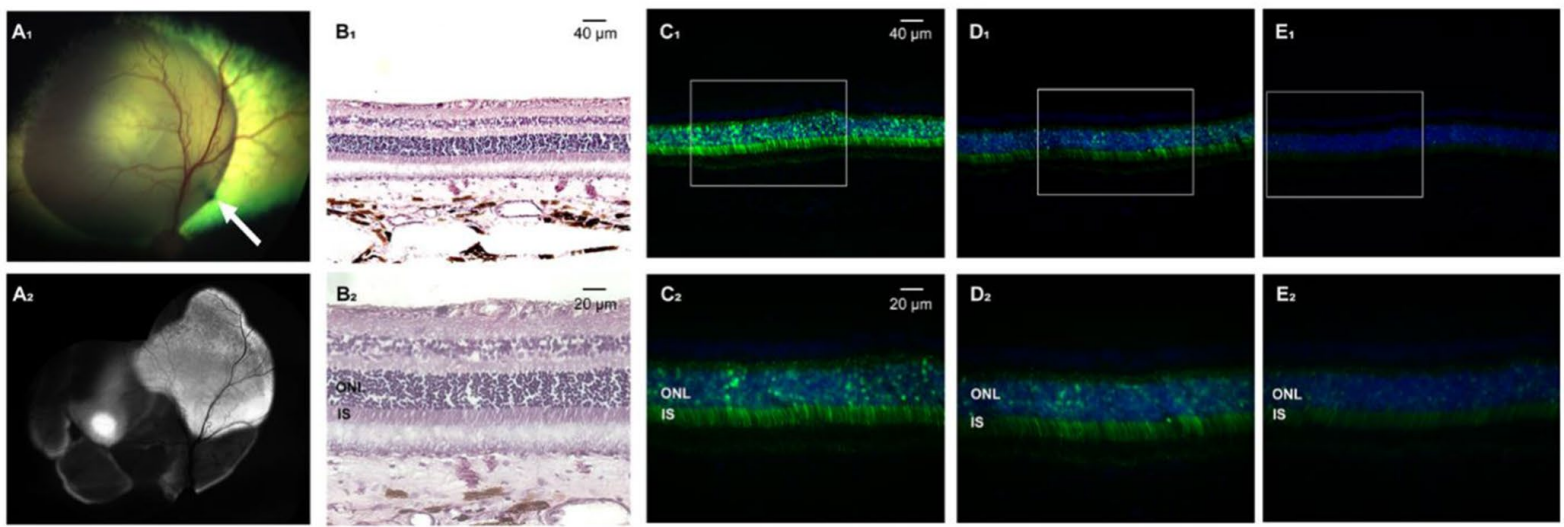
Successful subretinal injection of AAV2/5-GRK1-*GFP* with Orbit^®^ SDS device target photoreceptors in an adult WT dog. (**A**_1_) Fundus photograph taken immediately post-injection in dog ID CKCCRY, with the injection site visible as a dark tapetal lesion in the mid-tapetum (white arrow). (**A**_2_) Blue light autofluorescence cSLO composite showing GFP fluorescence with heterogenous intensity in the bleb area at 5 weeks post-injection. (**B**_1− 2_) H&E-stained sections showing healthy retinal layering within the bleb area at 20x (**B**_1_) and 40x (**B**_2_) magnification. (**C**_1− 2_ - **E**_1− 2_) 20x and 40x magnification sections taken in different areas within the treated area showing varied levels of native GFP fluorescence. Fluorescence appears especially concentrated within the outer nuclear layer (ONL), and inner segments (IS)

Using the same surgical approach as described above, successful SRIs of AAV2/5-GRK1-*GFP* (0.15 mL at 1.51 × 10^11^ vg/mL) were achieved in the tapetal region of 4 adult WT dogs. Resistance was felt when extending the microneedle through the choroid, which required, in several eyes, multiple attempts to enter the SRS (Table 1). In one eye (ID: CKCCPG), the microneedle was seen perforating the overlying neuroretina, which was confirmed at 5 weeks PI by non-invasive OCT imaging (data not shown). Following subretinal injection, the retinal bleb was resorbed and the neuroretina reattached within 24–48 h. In vivo detection of GFP expression by cSLO imaging at 5 weeks (Fig. 2 A_2_) and 9 weeks PI revealed a heterogenous pattern of fluorescence in all 4 injected eyes which was confirmed by detection of varying levels of native GFP fluorescence on retinal cryosections within the treated area (Fig. 2 C_1 − 3_, D_1 − 3_). No clinical (Fig. 2 A_1 − 2_), nor histological (Fig. 2 B_1 − 2_) signs of retinal alterations/inflammation were seen in any of the eyes.

To determine whether the SRS can be reached via this surgical approach in a dog with advanced photoreceptor loss we used the Orbit^®^ SDS clinical-grade device to deliver the same vector and dosage, into the tapetal region of an adult mutant *PDE6B*^*−/−*^ dog (ID: 2353) with advanced retinal degeneration. A subretinal bleb was successfully achieved (Suppl. Figure 3 A_1− 3_, B) and post-surgical analyses of videos, OCT and OCTA scans confirmed that the neuroretina had not been inadvertently perforated at the site of injection. A heterogenous pattern of GFP fluorescence was seen within the bleb area by cSLO imaging which may be attributed to the advanced stage of disease and severe loss of rods at that age (Suppl. Figure 3 C, D).

### Orbit® SDS device can be successfully used for SRI in juvenile WT eyes

**Fig. 3:**
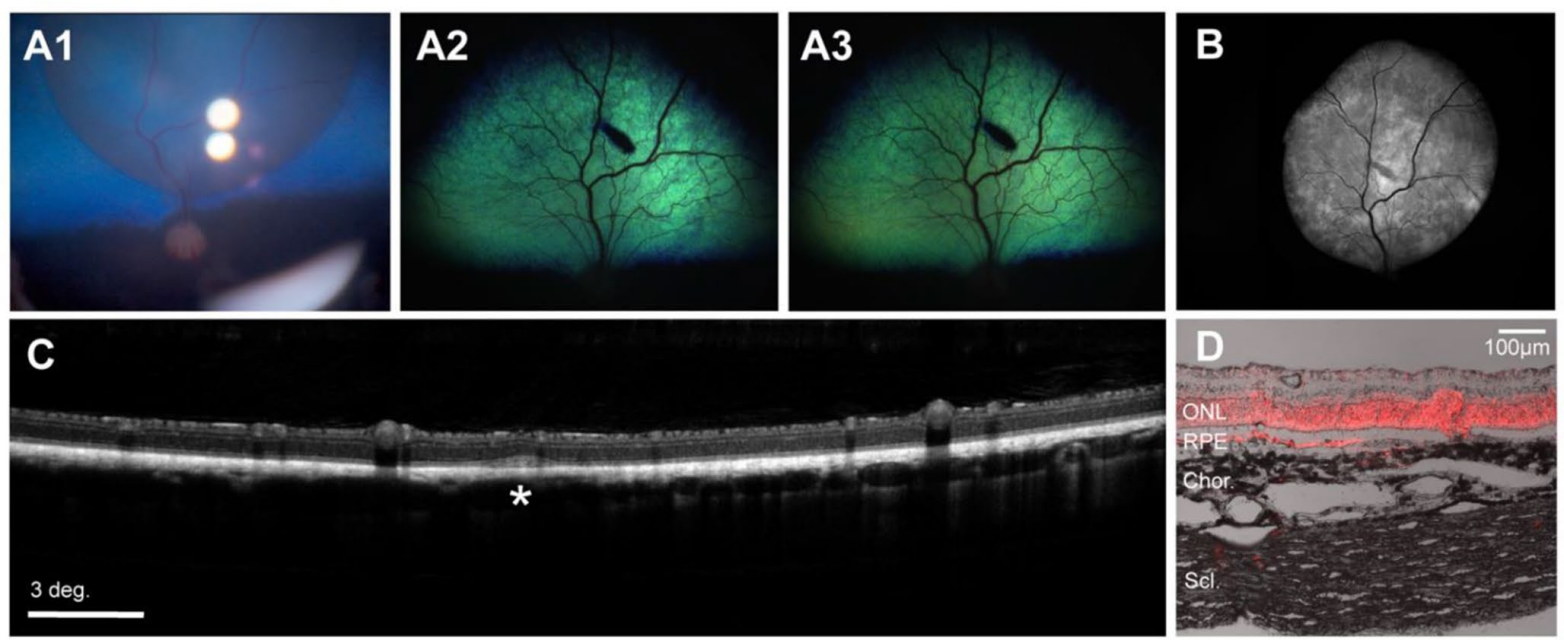
Illustration of successful subretinal injection with the Orbit^®^ SDS device in a 12-week-old pup. (**A**_1_) Intraoperative fundus photography showing a newly created subretinal bleb in dog ID 1168382 injected with AAV2/5-CBA-*tdTomato*-WPRE. (**A**_2− 3_) Fundus photographs at 5 weeks (**A**_2_) and 9 weeks (**A**_3_) post-injection (PI) show a healthy fundus with a black tapetal lesion where the needle penetration occurred. (**B**) Blue light autofluorescence cSLO composite image showing fluorescence in the subretinally-injected area at 9 weeks PI. (**C**) The retina shows normal lamination with no signs of toxicity detectable by OCT. At the site of microneedle penetration into the subretinal space focal disruption of the IS/OS layering can be seen (white asterisk). (**D**) Positive native tdTomato fluorescence in the ONL and RPE of the treated retina with scant amounts visible in the sclera within the cannula path close (∼ 1 mm) to the puncture/injection site

Following testing of the Orbit^®^ SDS clinical-grade device in 9 adult canine eyes we attempted to use this device design in 3 eyes of juvenile (19 week-old) dogs. While SRI in the non-tapetal region was successfully achieved after the 1st or 2nd microneedle advancement, entering the SRS in the tapetal region of juvenile and adult eyes often required multiple microneedle advancements and/or repositioning of the cannula. Difficulty at engaging the microneedle though the tapetum prompted us to develop and test novel Orbit^®^ SDS prototypes that were configured to have different cannula tips (troughed, un-troughed, or wedge-shaped) and microneedles with varying distal curvatures (low, mid, high) (Suppl. Figure 1 C). The purpose of these design changes was to accommodate the dogs’ smaller globe size and provide a more perpendicular needle approach angle with the tapetum. These were tested in 5 WT juvenile dogs (ages 10–19 weeks, total 7 eyes). Injections were successful in six eyes, while one attempt failed (Table 1). Notably, in 5 eyes, injection with either Orbit^®^ SDS prototype 1 (3 eyes) or Orbit^®^ SDS prototype 2 (2 eyes) was successful in the tapetal region with only one single microneedle advancement into the subretinal space (Fig. 3). The ease of access grade for each device design (mean, mode) was as follows: Orbit^®^ SDS 2nd Gen (1.67, 1); Orbit^®^ SDS Proto1 (1, 1); Orbit^®^ SDS Proto2 (1, 1), Orbit^®^ SDS Proto3 (4, 4), Orbit^®^ SDS Proto4 (4, 4); Orbit^®^ SDS NHP (4, 4). The subretinal space was successfully reached in 19 of the 22 devices tested, resulting in a total device delivery success rate of 86%.

Surgically-induced lesions that were noted included suspected retinotomy, minor subretinal bleeding, and scleral scarring. At the time of microneedle advancement, retinal perforation of the neuroretina was suspected in 10 out of 20 eyes. Subretinal bleeding was not always visible immediately after injection but became apparent 24 h post-SRI. Subretinal hemorrhage was seen after SRI in 10 eyes and was resorbed within 1–3 weeks PI. These hemorrhages were observed regardless of whether an iatrogenic retinal perforation was suspected- or whether anterior chamber paracentesis had been performed. In affected eyes, the retinal blebs fully reattached by one week PI. At the site of the sclerotomy and cannula insertion, a sharp deviation in scleral curvature was found by OCT and localized retinal damage was seen in 7 eyes (ages 10–19 weeks at SRI) by fundus photography and cSLO (Suppl. Figure 4). These peripheral lesions remained static between 5 and 9 weeks PI.

### Assessment of surgically-induced choroidal neovascularization

**Fig. 4:**
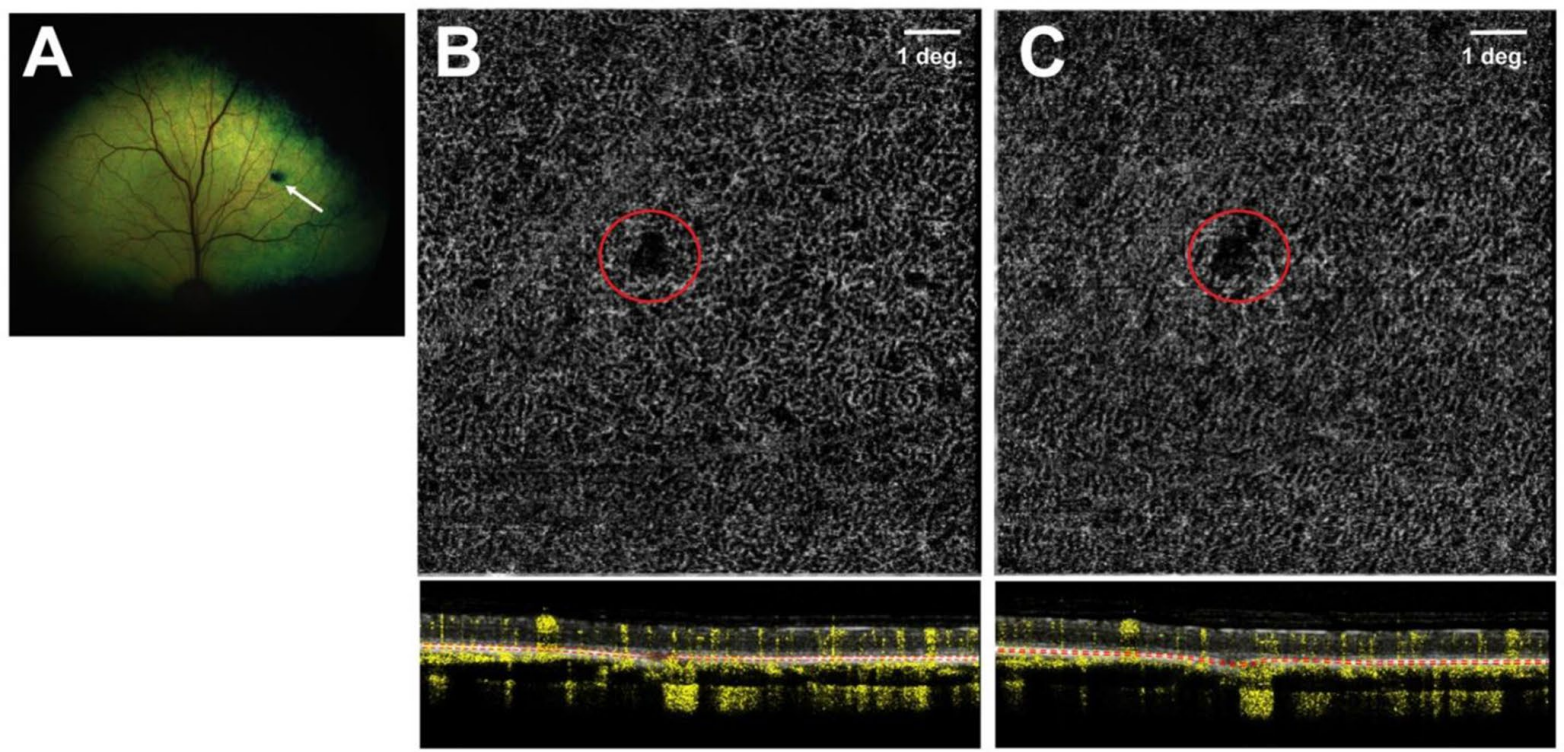
Absence of choroidal neovascularization (CNV) following use of the Orbit^®^ SDS device in dogs. (**A**) Fundus photograph taken at 5 weeks post-injection (PI) in an adult WT dog (ID: N361) showing a dark lesion at the site of needle penetration (white arrow) through the tapetum lucidum. (**B**-**C**) No CNV lesions were seen by OCT angiography (OCTA) in the choriocapillaris at the site of needle puncture (red circle) either at 5 weeks (**B**) or 9 weeks (**C**) PI

All 18 successfully injected eyes were assessed by OCTA for the development of potential CNV lesions originating from the choriocapillaris at the site of microneedle puncture. In 14 of these eyes, OCTA imaging showed no evidence of CNV lesions at either the 5-week or 9-week PI timepoints (Table 3). In the 4 eyes from two dogs (IDs: N356, SSA-14), OCTA imaging was inconclusive due to poor resolution caused by uveitis. At microneedle puncture sites, a persistent limited and localized defect in the choriocapillaris was often seen but without signs of neovascular proliferation (Fig. 4).

**Table 3:** Assessment of choroidal neovascularization (CNV) lesions post-subretinal injection with Orbit^®^ SDS device

Dog ID	Vector	Eye	CNV lesions at 5wks PI	CNV lesions at 9wks PI
CKCCPG	AAV2/5-GRK1-*GFP*	OD	None	None
CKCCRD	AAV2/5-GRK1-*GFP*	OD	None	None
CKCCRY	AAV2/5-GRK1-*GFP*	OD	None	None
CKCCPB	AAV2/5-GRK1-*GFP*	OD	None	None
2353	AAV2/5-GRK1-*GFP*	OD	None	N/A**
N356	AAV2/5-CBA-TdTomato-WPRE	OD	Unable to assess*	N/A**
		OS	Unable to assess*	
SSA-14	AAV2/5-CBA-TdTomato-WPRE	OD	Unable to assess*	None
		OS	Unable to assess*	None
N360	AAV2/5-CBA-TdTomato-WPRE	OD	None	None
		OS		
1054106	AAV2/5-CBA-TdTomato-WPRE	OD	None	None
			None	None
N361	AAV2/5-CBA-TdTomato-WPRE	OD	None	None
		OS	None	None
1168382	AAV2/5-CBA-TdTomato-WPRE	OD	None	None
1180608	AAV2/5-CBA-TdTomato-WPRE	OD	None	None
1180560	AAV2/5-CBA-TdTomato-WPRE	OD	None	None
		OS	None	None

* Due to severe panuveitis

** Experiment ended at 6 weeks PI due to severe panuveitis noted at 5 weeks PI

### AAV transgene expression is found in the sclera and choroid along the path of the Orbit® SDS cannula

**Fig. 5:**
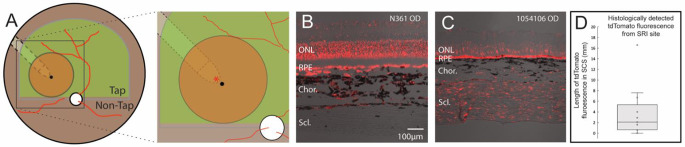
Viral vector transgene (tdTomato) expression in the choroid and sclera along the SCS tunneling path of the Orbit^®^ SDS cannula. (**A**) Schematic of a canine fundus showing: a subretinal bleb (orange disc) in the tapetal (Tap) region, the location of suprachoroidal tunneling path followed by the Orbit^®^ SDS cannula (dotted line), and the site of microneedle puncture into the subretinal space (black dot). Red asterisk indicates approximate location of retinal sections shown in (**B**) and (**C**). (**B**) Evidence of native tdTomato fluorescence in the choroid of the mid-peripheral tapetal fundus along the cannula path close (∼ 1 mm) to the puncture/injection site and, (**C**) in the sclera in the central non-tapetal fundus along the cannula path close (∼ 1 mm) to the puncture/injection site. (**D**) Box-whisker plot showing range of distances over which tdTomato was found in the SCS from the site of injection

Distribution of AAV in the choroid/sclera following SRI was assessed in 14 eyes across 8 normal dogs (Table 4). Native tdTomato fluorescence was strongest in the sclera (Fig. 5C) and to a lesser extent in the choroid adjacent to the injection site (Fig. 5B) and intensity of the signal was decreased at distances further away from the injection site. Fluorescence was detected primarily along the SCS path through which the cannula had been inserted, and distances of detectable tdTomato fluorescence away from the injection site ranged from 0 mm to > 16.6 mm, with a median of 2.1 mm (IQR: [0.65, 5.35]) (Fig. 5C).

**Table 4:** Viral vector transgene (tdTomato) expression in the choroid and/or sclera

Dog ID	Age at SRI (wks)	Eye	tdTomato fluorescence in sclera/choroid	Length of expression (mm)
N356	72	OD	Yes	> 16
		OS	Yes	1.6
SSA-14	61	OD	Not detected	0
		OS	Yes	1.7
N360	19	OD	Yes	0.7
		OS	Yes	0.6
1054106	19	OD	Yes	> 16.6
N361	19	OD	Yes	6.7
		OS	Not detected	0
1168382	12	OD	Yes	2.2
1180608	10	OD	Yes	2.9
1180560	10	OD	Yes	7.6
		OS	Yes	2.1

### Clinical and histological signs of inflammation following AAV delivery with Orbit® SDS device are dose dependent

Clinical signs of ocular inflammation, including aqueous flare, cells in the anterior chamber, miosis, chorioretinitis, intravitreal hemorrhage and partial retinal detachment were observed in 4 eyes of 2 dogs (IDs: SSA-14, N356) that had been treated with viral vector titers of 5 × 10^11^ and 1 × 10^12^ vg/mL, respectively. These signs were first detected at 5 weeks PI, 5–7 days following the cessation of oral prednisone and persisted for the rest of the in-life period. H&E staining of retinal sections revealed the presence of lymphoplasmacytic infiltrates in the neuroretina and choroid, and immunohistochemical analysis confirmed the activation of innate (Iba1, CD18) and adaptive (CD4, CD8, CD20) cellular immune responses (Table 5).

**Fig. 6:**
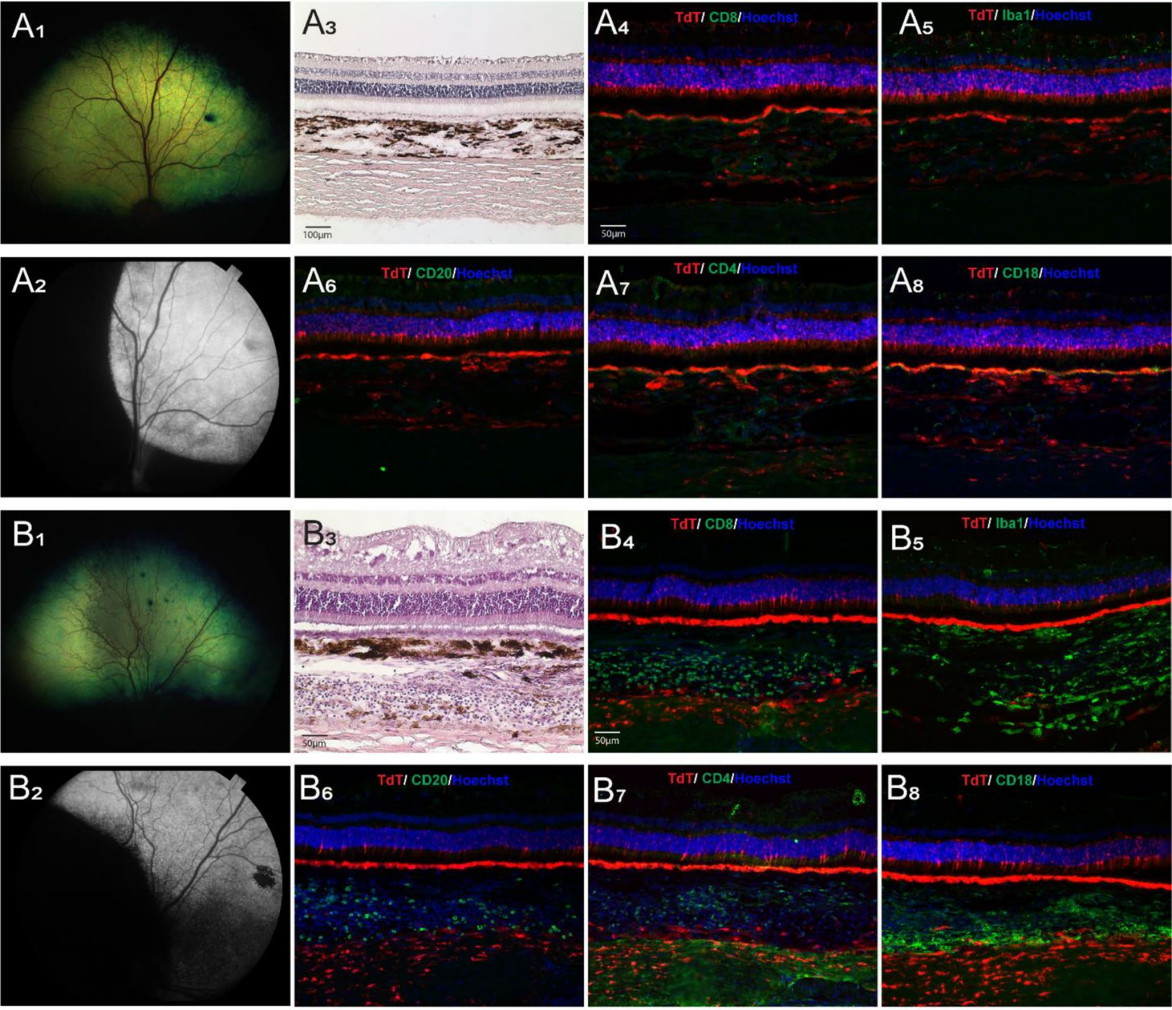
Clinical and histomorphological assessment of ocular inflammation in eyes injected with AAV2/5-CBA-*tdTomato*-WPRE (titer: 1.5 × 10^11^ vg/mL) via Orbit^®^ SDS device. (**A**_1− 8_) Illustration of a case (dog ID: N361-OD) with potent expression of tdTomato and no evidence of clinical nor histological/IHC signs of inflammation. Fundus photograph showing normal retinal appearance (**A**_1_). Fundus photograph (B&W) showing tdTomato fluorescence in the injected area (**A**_2_). Histological (H&E stain) showing normal retinal, choroidal and scleral structure (**A**_3_). IHC labeling of cell markers (CD4, CD8, CD18, CD20, and Iba1, in green) and native tdTomato fluorescence (in red) showing absence of inflammatory cell infiltration in the retina, choroid, and sclera (**A**_4− 8_). (**B**_1− 8_) Illustration of a case (dog ID: 1054106-OD) with potent expression of tdTomato, and evidence of mild clinical but prominent histological/IHC signs of inflammation. Fundus photograph showing altered tapetal reflectivity in the area that was subretinally injected (**B**_1_). Fundus photograph (B&W) showing tdTomato fluorescence in the injected area (**B**_2_). Histological (H&E stain) showing prominent cellular infiltration in the choroid (**B**_3_). IHC labeling of cell markers (CD4, CD8, CD18, CD20, and Iba1, in green) and native tdTomato fluorescence (in red) showing a mixed population of inflammatory cellular infiltrates in the choroid (**B**_4− 8_)

Among the 6 dogs (8 eyes) injected with a lower titer (1.5 × 10^11^ vg/mL), clinical and histological signs of chorioretinitis were seen in both eyes of one dog (ID: N360) at 9 weeks PI. In another animal that was only injected in one eye (ID: 1054106), mild funduscopic signs of tapetal hyporeflectivity were seen at 9 weeks PI in the injected area (Fig. 6, B_1_), and histological assessment confirmed choroidal inflammation (Table 5; Fig. 6, B_4 − 8_). No inflammation was detected in the remaining eyes, including the single eye (ID: N361-OS) injected with the lowest (5 × 10^10^ vg/mL) titer (Table 5).

**Table 5:** Summary of clinical and histological signs of inflammation in dogs with Orbit^®^ SDS and prototypes

Dog ID	Age at SRI (wks)	Eye	AAV2/5-CBA-TdTomato-WPRE Titer (vg/mL) / Vol (mL)	Clinical Inflammation	Histological Inflammation (severity)	Inflammatory cells
N356	72	OD	1 × 10^12^ / 0.18	+++	Retina (++), Choroid (+++)	CD4, CD8, CD20, Iba1, CD18
		OS	1 × 10^12^ / 0.18	+++	Retina (+), Choroid (+++)	CD4, CD8, CD20, Iba1, CD18
SSA-14	61	OD	5 × 10^11^ / 0.18	+++	Retina (+++), Choroid (+++)	CD4, CD8, CD20, Iba1, CD18
		OS	5 × 10^11^ / 0.18	+++	Retina (+++), Choroid (+++)	CD4, CD8, CD20, Iba1, CD18
N360	19	OD	1.5 × 10^11^ /0.15	+++	Retina (+++), Choroid (+++)	CD4, CD8, CD20, Iba1, CD18
		OS	1.5 × 10^11^ /0.15	++	Retina (++), Choroid (++)	CD4, CD8, CD20, Iba1, CD18
1054106	19	OD	1.5 × 10^11^ /0.15	+	Choroid (+++)	CD4, CD8, CD20, Iba1, CD18
N361	19	OD	1.5 × 10^11^ /0.15	None	None	None
		OS	5 × 10^10^ /0.15	None	None	None
1168382	12	OD	1.5 × 10^11^ /0.15	None	None	None
1180608	10	OD	1.5 × 10^11^ /0.15	None	None	None
1180560	10	OD	1.5 × 10^11^ /0.10	None	None	None
		OS	1.5 × 10^11^ /0.10	None	None	None

## Discussion

Access to the SRS remains a delicate surgical procedure for the delivery of cell and gene therapy products. Complications associated with the standard transvitreal SRI technique have prompted the development of new routes of delivery that circumvent the need for a vitrectomy and a retinotomy [32, 33]. The use of the Orbit^®^ SDS device is one of the latest approaches that enables easy access to the SRS without breaching the integrity of the neuroretina [34]. To determine whether this device could be used for future studies in canine models of IRD, we evaluated here the surgical feasibility, its challenges and its complications in 20 canine eyes of various ages. Additionally, we provide the first evidence of choroidal and scleral AAV transgene expression following successful SRI with the Orbit^®^ SDS device. Finally, we observed clinical and histological signs of inflammation that appear to be AAV dose dependent.

### Surgical considerations with the use of Orbit® SDS device in canine eyes

When first testing ex vivo the device on adult canine eyes, we were unable to extend the cannula into the SCS when the sclerotomy was performed 6 mm posterior to the limbus as recommended in pig and human eyes. No such problem was encountered with a more posterior scleral incision (~ 10 mm from the limbus). The cannula could be easily advanced towards the posterior pole of the globe in all 20 eyes. This contrasts with the difficulty frequently encountered at engaging the cannula in the SCS of pig eyes likely due to dense choroido-scleral adhesions [34], and suggests that similar dense connective tissue may preclude separation of the uveal and scleral tunics in the anterior portion of the canine globe. Histological characterizations of the choroid across animal species consistently describe the presence of five sublayers of which the suprachoroidea is the most external [35–37] Composed of elastic reticular fibers, fibroblasts and melanocytes, the suprachoidea’s function is to maintain the choroid attached to the sclera. Loco-regional and/or species-specific variation in these tensile properties may explain resistance to cannulation with devices such as the Orbit^®^ SDS or incomplete spread throughout the SCS following suprachoroidal injections [38].

Another anatomical consideration specific to the canine eye that had to be considered to optimize the surgical approach is the presence in the anterior sclera of a deep intrascleral venous plexus and the venous circle of Hovius [39, 40] located ~ 3–5 mm posterior to the limbus in adult eyes with an axial globe length of ~ 21–22 mm [41]. This vascular plexus participates in the conventional aqueous humor outflow from the corneoscleral trabeculum. Performing the sclerotomy ~ 10 mm behind the limbus avoided the major intrascleral vessels, though cauterization was still needed. In the smaller eyes of 10–12 week-old dogs (axial globe length: ~ 16–17 mm) [20], the sclerotomy was done 6 mm posterior to the limbus.

A significant difference in surgical experience in canine eyes versus in ex vivo pig and human eyes was the difficulty initially encountered at advancing the microneedle through the choroid when using the clinical-grade Orbit^®^ SDS device (2nd generation) particularly in younger dogs with smaller globes. In several dogs this required multiple attempts by extending and retracting the needle until it finally punctured through the choroid/RPE and entered the SRS. This difficulty was felt when attempting SRI injections in the tapetal fundus but not in the non-tapetal area. Dogs like many carnivores (but unlike pigs and primates) have a multilayered choroidal structure composed of rectangular-shaped cells that contain reflective material. This tapetum lucidum is thought to play the role a biological light reflector that provides adaptation to vision under low illumination [42]. In the dog, these 9–15 tightly packed layers of cells likely constituted a physical barrier to the extension of the microneedle, and as a result prototypes with more aggressive needle curvatures were tested. Improved advancement of the microneedle through the tapetum was observed with devices that had either a high or mid curvature microneedle and a troughed cannula (Suppl. Figure 1). The high and mid curvature microneedles have a more perpendicular angle of approach to the tapetum than the clinical grade and low curvature devices, increasing tissue strain during needle extension allowing for easier needle puncture.

Finally, the presence in the canine eye of a third eyelid (also called nictitating membrane) limits the placement of the sclerotomy and cannulation of the suprachoroidal space to the superior and temporal quadrants. Through such a port of entry, it was possible to extend the cannula into the inferior fundus and make a subretinal injection in the non-tapetal region.

In summary although the canine eye presents anatomical challenges (anterior choroido-scleral adhesions, deep intrascleral venous plexus, choroidal tapetum, and nictitating membrane) that are not encountered in the human eye, by adjusting the placement of the Orbit^®^ SDS device to circumvent these anatomical structures one can successfully achieve a subretinal injection.

### Surgical complications associated with the use of the Orbit® SDS device in dogs

Although more aggressive needle curvatures improved the success rate at entering the SRS on the first attempt, inadvertent retinotomies still occurred. Overall, injections in 10 out of 20 eyes were suspected to have resulted in a retinotomy (Table 1). Despite this, all retinotomies created by the Orbit^®^ SDS device were small and only detectable through careful post-surgical analysis of surgical videos and *en face* OCT at injection sites. Indeed, linear volumetric OCT scans did not reliably reveal the presence of retinotomies caused by the Orbit^®^ SDS microneedles. The incidence of retinal perforations in dogs in this study was higher than that reported in minipigs (4 out of 27) and in human patients with geographic atrophy (0 out of 21) that had umbilical tissue-derived cells subretinally injected using a precursor of the Orbit^®^ SDS device [18, 19]. This difference may be explained by the presence of a tapetum in the dog and the inhomogeneity of the tapetal and retinal tissue layers. Once tapetal tissue rupture occurs, the strain energy is released into the more delicate retina resulting in a puncture, i.e. a retinotomy [43].

In 10 out of 18 canine eyes that received an in vivo SRI, a subretinal hemorrhage was seen immediately after surgery and, occasionally, 24 h later. This bleeding was likely choroidal in origin as no damage to the retinal vasculature was noted by *en face* OCT. Retinal hemorrhages following SRI with a similar device have also been observed in pigs (1 out of 27) and humans (4 out of 21 patients) [18, 19].

No rapid flattening of the bleb was observed in any of the 20 canine eyes immediately after the SRI, suggesting that retraction of the microneedle from the SRS and then of the cannula from the SCS, did not cause any significant acute reflux and loss of the delivered viral vector suspension. This aligns with previous work evaluating the delivery efficiency of Orbit^®^ SDS where bleb retention and volume delivered subretinally was quantitatively measured with microscope integrated OCT [34].

Examination at 5- and 9-weeks post SRI of the far peripheral fundus by indirect ophthalmoscopy, fundus photography, and cSLO revealed a localized focal lesion in the quadrant where the Orbit^®^ SDS device was placed in 7 out of 18 eyes. This could also be seen by OCT imaging that revealed scleral warping and was confirmed by histology that showed occasionally some retinal damage. This scleral warping which was seen exclusively in dogs that received SRI at a young age (10–19 weeks) may be due to lower scleral stiffness in younger individuals than in adults [44]. These lesions were found at the site of the sclerotomy where cauterization of intrascleral vessels was performed before the introduction of the Orbit^®^ SDS cannula into the SCS. Electrocoagulation may have caused focal scleral shrinkage and heat-induced damage to the underlying choroid and retina. As the animals were terminated 9 weeks post SRI, long-term effects of scleral deformation could not be evaluated. As this was a very focal lesion limited to the site of the sclerotomy that was performed posterior to the pars plana, we do not expect it could cause any damage to the lens zonules, corneal deformation that would lead to astigmatism, nor any detrimental impact on eye growth. It is important to highlight that electrocautery is not required when using the Orbit^®^ SDS device in human eyes.

Laser photocoagulation-induced rupture of Bruch’s membrane is commonly used to trigger choroidal neovascularization in animal models [45]. Thus, an obvious concern with the mechanical disruption of Bruch’s membrane and lesions to the choriocapillaris that occur as a result of the advancement of the Orbit^®^ SDS microneedle into the SRS, is the potential for iatrogenic development of CNV. Imaging of the choriocapillaris that was performed up to 9 weeks post SRI did not reveal CNV lesions at the site of microneedle puncture in any of the 12 eyes in which OCTA could be performed. Although CNV does not occur spontaneously in dogs, a single study to this date has shown that vascular lesions of CNV can be seen by fluorescein angiography within 4 weeks after laser photocoagulation in Beagle dogs [46]. Thus, this study provides further support for the safety of this route of delivery to the SRS in normal retinas. While no CNV was observed at 5-week post SRI in the single mutant *PDE6B*^*−/−*^ dog included in this study, assessment at 9 weeks post SRI was precluded by the loss of ocular transparency caused by the severe panuveitis that occurred in that eye. Thus, determining whether CNV lesions could be triggered within the proinflammatory environment of a diseased/degenerating retina will need further investigation.

### Potential reflux of AAV solution in the SCS

A limitation of transvitreal SRI is the potential for reflux into the vitreal cavity of the delivered cell or gene therapies that can trigger adverse effects (e.g. formation of epiretinal membranes, intraocular inflammation) and reduce the dose intended to be delivered subretinally [24, 47]. This reflux can occur during injection or after the formation of a subretinal bleb. Although a two-step approach, where a small volume of BSS is first injected into the SRS, followed by further expansion of the bleb via the same retinotomy with the viral vector solution, reduces reflux, it still results in up to 1.3% of the subretinal dose being refluxed into the vitreal cavity [48]. Even such a small amount of a highly concentrated dose of viral vector can trigger inflammation, as the vitreal cavity is less immune-privileged than the SRS. Surgeon tremors during the injection and drifting of the subretinal cannula while engaged into the SRS can lead to iatrogenic enlargement of the retinotomy and increased vitreal reflux [1]. Access to the SRS via a suprachoroidal approach was developed precisely to avoid the formation of a retinotomy and vitreal reflux particularly in vitrectomized eyes. In a recent study conducted in minipigs, the use of microscope-integrated OCT (MIOCT) revealed that the mean subretinal volume was 82% of the intended injected volume [34]. As authors reported not seeing any perforation of the neuroretina that could have led to vitreal reflux, they suggested that the loss may have occurred through reflux into SCS. Using a viral vector carrying a fluorescent reporter gene (tdTomato) under control of a ubiquitous promoter, this current study showed that expression of the transgene was found in 11 out of 13 eyes along the path used to introduce the Orbit^®^ SDS cannula in the SCS. As MIOCT and estimation of bleb volume was not performed, we could not unequivocally conclude that the presence of viral vector in the SCS was caused by reflux rather than by leakage of remaining AAV solution present in the cannula upon retraction of the device. Leakage of AAV into the SCS has the potential for triggering an immune-mediated response as the SCS, unlike the SRS, is not an immune-privileged site.

Clinical signs of ocular inflammation were seen in 4 dogs (7 eyes) injected with AAV2/5-CBA-*tdTomato*-WPRE and confirmed histologically with the presence of infiltrating T and B cells as well as microglia/macrophages in the neuroretina and choroid (Table 5). All four eyes injected with a titer ≥ 5 × 10^11^ vg/mL had severe inflammation. Of the 8 eyes injected with a titer of 1.5 × 10^11^ vg/mL, only 3 eyes had clinical and histological signs of inflammation. Inflammation was not correlated with the extent of tdTomato expression in the SCS (Tables 4 and 5). This study did not investigate whether the immune response was directed against the AAV5 capsid or the transgene (tdTomato) that was expressed in choroidal cells and scleral fibroblasts. Clinical relevance of an immune response towards tdTomato is limited as there are no gene therapy products for subretinal delivery to the human eye that carry such reporter gene. However, if the response was directed against the transgene, future use of a photoreceptor-specific promoter could attenuate the severity of this immune response.

## Conclusion

The Orbit^®^ SDS device can be successfully used to perform SRIs in juvenile and adult canine eyes. The choroidal *tapetum lucidum* in the dog constitutes a physical barrier that complicates microneedle engagement, especially in younger dogs. The use of devices with high-curvature microneedles circumvented this limitation yet often caused micro-perforation of the underlying neuroretina. However, the secure placement of the subretinal Orbit^®^ SDS microneedle allowed for greater control over the injection timing and volume delivered, and prevented inadvertent enlargement of the retinotomy that often occurs during transvitreal SRI. In many eyes, OCT imaging of the site of puncture several weeks post SRI did not reveal persistent retinotomies suggesting that these may have self-sealed. Reflux into the SCS was found in most eyes along the track of the cannula, yet ocular inflammation appeared to be dose-dependent, thus providing a potential dose range that may be safe. In summary, this device can be a good addition to the vitreoretinal surgeon’s arsenal of approaches for performing subretinal injections.

## Electronic supplementary material

Below is the link to the electronic supplementary material.

Supplementary Material 1

## Data Availability

The datasets generated during and/or analysed during the current study are available from the corresponding author on reasonable request.
