# Supplementary material for: Assessment of the Orbit® subretinal delivery system (Orbit® SDS device) and prototypes in adult and juvenile canine eyes
Source: Drug Deliv Transl Res. 2025 Aug 2;16(4):1285–300. doi: 10.1007/s13346-025-01929-2 (PMC12520015; doi:10.1007/s13346-025-01929-2)
Supplement: Supplementary file 1 — Supplementary Material 1 [file 13346_2025_1929_MOESM1_ESM.docx]

**Supplementary Figures**


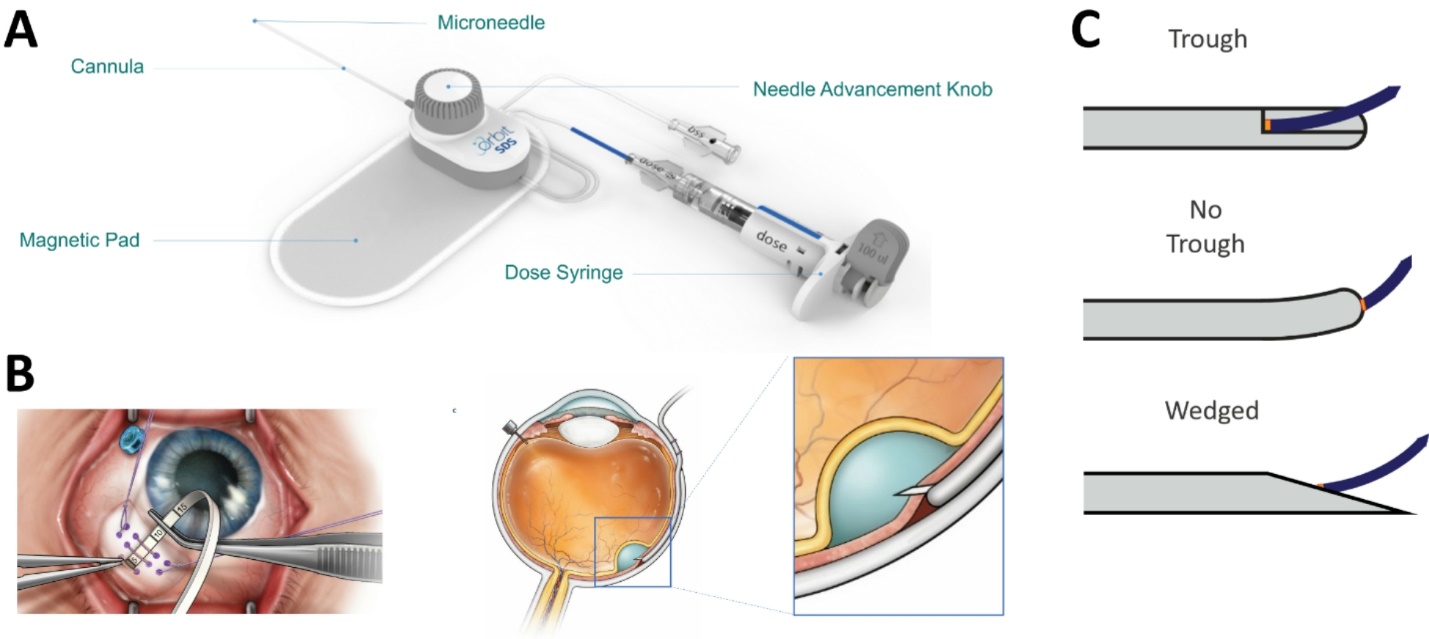


**Supplementary Fig. 1: Diagram of the Orbit® SDS device and modified cannulas and microneedles. (A)** The Orbit**®** SDS device is comprised of a main body that is secured to an adhesive magnetic pad. Attached to the device is a cannula with a microneedle that can be advanced out of its tip by adjusting the needle advancement knob. One fluid line connected to a BSS syringe and another fluid line connected to the dose syringe are separately attached to the device. **(B)** Diagram of SRI using Orbit**®** SDS device. The injection cannula is secured to the sclera via stay sutures and slipped through a sclerotomy into the suprachoroidal space. The microneedle can be advanced out of the cannula to inject solutions into the subretinal space. **(C)** Types of cannula tips and microneedles tested in different Orbit**®** SDS devices for this study.


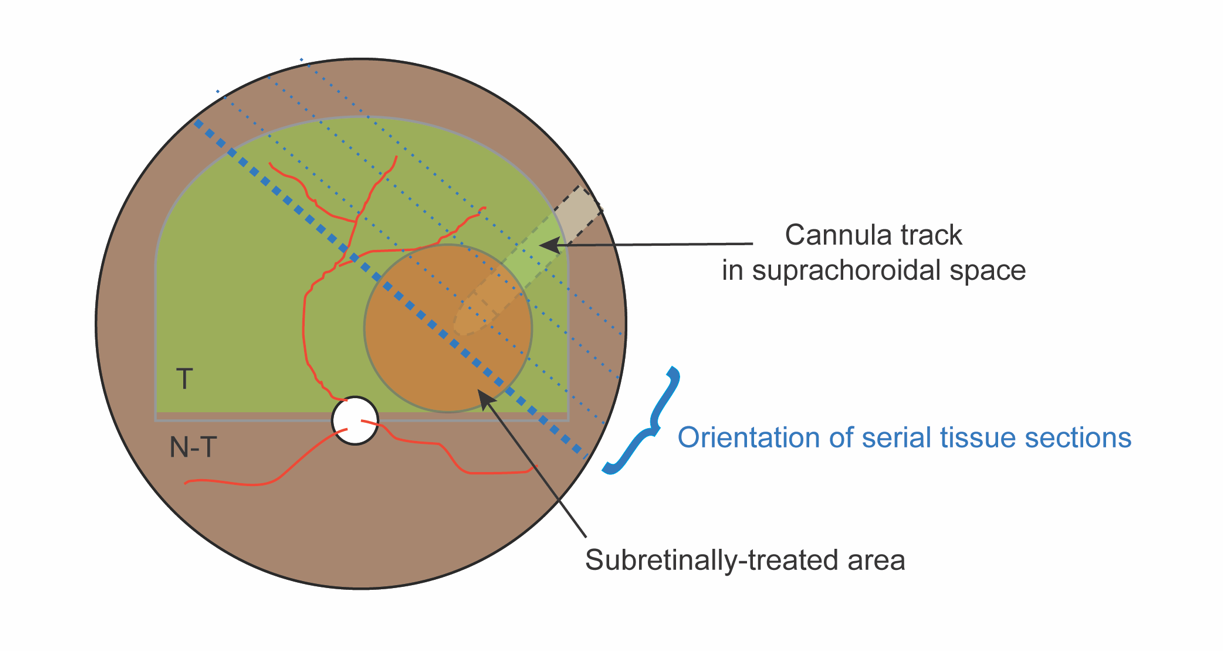


**Supplementary Fig. 2: Diagram of histological processing.** Paraformaldehyde-fixed posterior ocular cups were sectioned such that transverse cuts (blue dotted lines) were made along the path of the Orbit**®** SDS cannula. This was done so that tdTomato fluorescence could be assessed as sections were made further away from the needle penetration site.


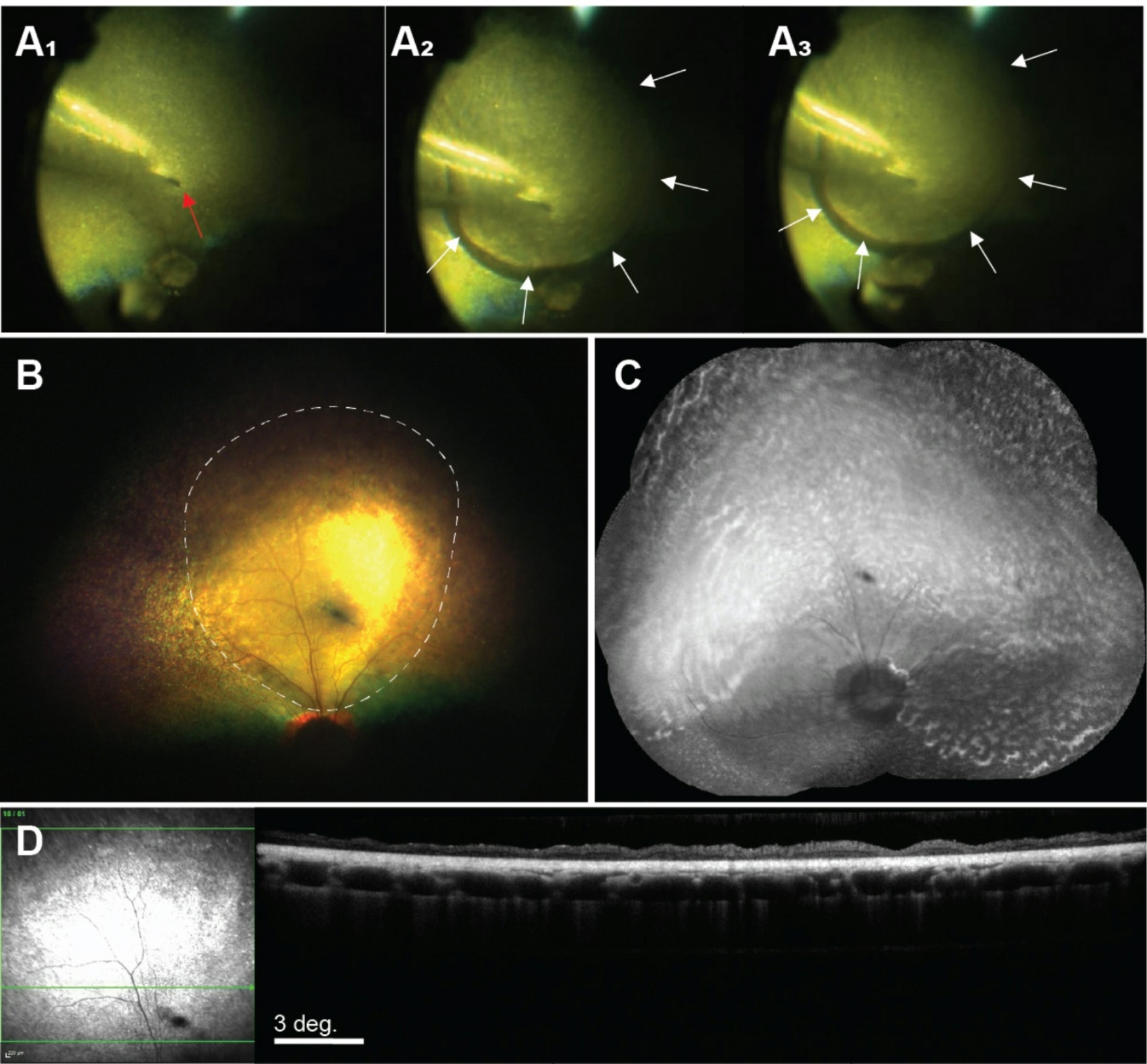


**Supplementary Fig. 3: Successful subretinal injection with Orbit® SDS device in a diseased mutant canine retina.** An adult rcd1 dog (ID: 2353) homozygous for a mutation in *PDE6B* was successfully injected with the AAV2/5-GRK1-*GFP* vector. **(A_1− 3_)** Peri-operative images extracted from a video recording show the Orbit ® SDS cannula entering and the needle protruding into the subretinal space (A_1_, red arrow), and the subsequent BSS bleb beginning to form (A_2− 3_, white arrows). **(B)** Fundus photograph immediately after successful SRI. The bleb is outlined as a white dotted line. **(C)** Composite montage of cSLO (Blue autofluorescence mode) images show heterogenous GFP fluorescence throughout a majority of the fundus at 5 weeks post-injection (PI), suggesting marked bleb expansion before natural reattachment of the neuroretina. **(D)** cSLO/OCT imaging 5 weeks PI throughout the previous bleb area shows a degenerated retina and no signs of toxicity.


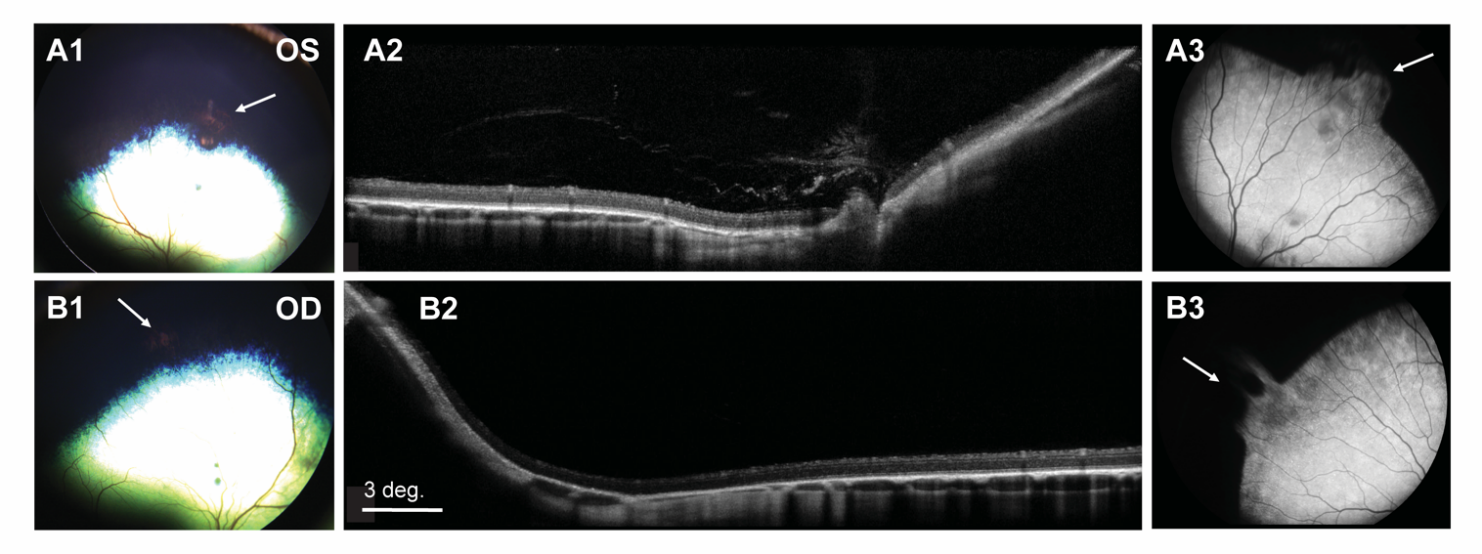
**Supplementary Fig. 4: Peripheral lesions seen at the site of Orbit** **^®^ SDS device insertion in some dogs.** **(A_1_, B_1_)** Fundus photographs in a WT dog (ID# N360) at 5 weeks post-injection of AAV2/5/CBA-*tdTomato*-WPRE shows hypopigmented lesions at the peripheral tapetal-non-tapetal border, where the Orbit**^®^** SDS cannula was inserted through the sclerotomy (white arrows). **(A_2_)** In some cases, there is minimal change in the scleral curvature but with some disruption of the retinal layering while in other cases, **(B_2_)** a sharp curvature of the sclera can be seen. **(A_3_, B_3_)** By fluorescence imaging, an out-pouching of the fluorescence (white arrows) detected in the bleb area can be seen extending out along the direction of the cannulation.

**Supplementary Table 1: List of primary antibodies used for immunohistochemistry.**

| **Antigen** | **Host** | **Source, Catalog #** | **Working conc.** | **Normal location** |
| --- | --- | --- | --- | --- |
| Dog CD4 | Rat Monoclonal IgG2a | Bio-Rad Antibodies, MCA1038GA | 1:50 | Helper T cell |
| Dog CD8 | Rat Monoclonal IgG1 | Bio-Rad Antibodies, MCA1039GA | 1:50 | Cytotoxic T cell |
| Dog CD18 | Mouse Monoclonal IgG1 | Leukocyte Antigen Laboratory, UC Davis, Sacramento, CA | 1:50 | Macrophages (blood/monocyte-derived) |
| Human CD20 | Rabbit Polyclonal IgG | Thermo Fisher scientific, PA5-16701 | 1:200 | B cells |
| Iba1 | Rabbit Polyclonal | FUJIFILM Wako Pure Chemical Corporation, 019-19741 | 1:500 | Microglia |

Appropriate fluorescent secondary antibodies (AlexaFluor™; A11006, A21121, A21206; Invitrogen) were used at 1:200 dilution.
